# Selective binding and transport of protocadherin 15 isoforms by stereocilia unconventional myosins in a heterologous expression system

**DOI:** 10.1038/s41598-022-17757-0

**Published:** 2022-08-12

**Authors:** Angela Ballesteros, Manoj Yadav, Runjia Cui, Kiyoto Kurima, Bechara Kachar

**Affiliations:** 1 Laboratory of Cell Structure and Dynamics, National Institute On Deafness and Other Communication Disorders, National Institutes of Health, Bethesda, MD 20892 USA; 2 Molecular Biology and Genetics Section, National Institute On Deafness and Other Communication Disorders, National Institutes of Health, Bethesda, MD USA; 3 Present Address: Molecular Physiology and Biophysics Section, NINDS, NIH, Bethesda, MD 20892 USA

**Keywords:** Protein transport, Motor proteins, Hair cell

## Abstract

During hair cell development, the mechanoelectrical transduction (MET) apparatus is assembled at the stereocilia tips, where it coexists with the stereocilia actin regulatory machinery. While the myosin-based tipward transport of actin regulatory proteins is well studied, isoform complexity and built-in redundancies in the MET apparatus have limited our understanding of how MET components are transported. We used a heterologous expression system to elucidate the myosin selective transport of isoforms of protocadherin 15 (PCDH15), the protein that mechanically gates the MET apparatus. We show that MYO7A selectively transports the CD3 isoform while MYO3A and MYO3B transports the CD2 isoform. Furthermore, MYO15A showed an insignificant role in the transport of PCDH15, and none of the myosins tested transport PCDH15-CD1. Our data suggest an important role for MYO3A, MYO3B, and MYO7A in the MET apparatus formation and highlight the intricate nature of MET and actin regulation during development and functional maturation of the stereocilia bundle.

## Introduction

The hallmark of hair cells, the sensory receptors of the inner ear is their mechanosensitive organelle, the hair cell bundle. This bundle consists of rows of actin-based protrusions of graded heights called stereocilia forming a characteristic staircase architecture. The hair cell bundle converts mechanical stimuli into electrical signals through a mechanoelectrical transduction (MET) apparatus, built around a protein filament, the tip link, that connects the tip of the shorter stereocilia to its closest taller neighbor^1^. This sophisticated architecture ensures the optimal gating of the MET channels located at the lower end of the tip link, when the stereocilia are deflected toward the taller stereocilia^2^. Remarkably, the MET molecular apparatus co-exists at stereocilia tips with the actin regulatory protein complex that sustains the stereocilia length differential, and there is increasing evidence of coordination between these two molecular machineries^3–5^.

Stereocilia tips can be up to a staggering 100 µm away from the hair cell body, which together with the restricted space between the stereocilia actin core and the plasma membrane makes it unlikely that molecular components efficiently traverse from cell body to stereocilia tips by simple diffusion. Like in other actin protrusions, active transport of molecular components in stereocilia is carried out by unconventional myosins, monomeric or dimeric myosins that transport cargoes along the surface of actin filament bundles^6^. The unconventional myosins MYO3A, MYO3B, and MYO15A have been implicated in transporting different actin-regulatory and scaffolding protein cargo to stereocilia tips^7–9^, but little is known about the transport of the MET components to stereocilia tips. Mutations in the MYO3A, MYO3B, MYO7A, and MYO15A genes have been implicated in different forms of inherited hearing loss through specific disruption of stereocilia structure and its normal mechanosensitivity^10–17^, suggesting a role for these myosins in stereocilia architecture and MET. The appearance of MYO3A in auditory stereocilia coincides with the onset of MET^18^, suggesting a role for MYO3 in the transport of the MET apparatus. Additionally, MYO7A localize to the stereocilia tips and has been implicated in regulating stereocilia structure and MET function^19–21^. Stereocilia myosins, like all myosins, typically share a similar motor domain but differ in their cargo-binding tail domain^22^. Because of the multitude of cargo and myosin tail domains, we hypothesize that stereocilia myosins selectively transport MET components to stereocilia tips but leave open the possibility for partial complementary functions and redundancy. Sorting out the pairing of motor and cargo is fundamental to understand the trafficking and selective targeting of stereocilia tip components, including the MET components that are essential for hearing and balance. How MET components are targeted to the tips of a specific stereocilia row in the staircase is key to understanding the mechanisms of MET complex assembly as well as an alluring cellular biology question. A key component of the MET complex is protocadherin 15 (PCDH15), which makes up the lower portion of the tip link that mechanically gates the MET channel^23,24^. Hair cells express three isoforms of PCDH15 (PCDH15-CD1, -CD2, and -CD3) that share a common extracellular domain but differ in the C-terminal region of their cytoplasmic tails^25^. The expression levels, localization, and role of each isoform is not fully understood but several studies have shown partial redundancy within the three PCDH15 isoforms during development with the CD2 isoform being essential for proper MET function in mature auditory hair cells^26–28^.

Studying transport of molecular components to the MET site at stereocilia tips is challenging because of the intricate relationships between molecular motors, actin regulation, and mechanosensitivity^7,9,19,20,29,30^. Perturbation of virtually any stereocilia component can either be masked by redundancy in function or rapidly cascade into disruption of hair bundle structure and function. Here we investigate the ability of myosins 3A, 3B, 7A, 10, and 15A, the prevalent myosins found in stereocilia, to individually transport the three main PCDH15 isoforms along filopodial actin protrusions in model cultured cells. We show that MYO3A and MYO7A show selective binding and transport of two distinct PCDH15 isoforms along actin protrusions. We argue that the selective transport of PCDH15 isoforms by stereocilia myosins is part of the dynamic mechanisms of spatiotemporal positioning of these key inter-stereociliary links and MET components to their functional location in the stereocilia.

## Results

### Dynamic localization of stereocilia myosins and PCDH15 during hair cell bundle development

**Figure 1:**
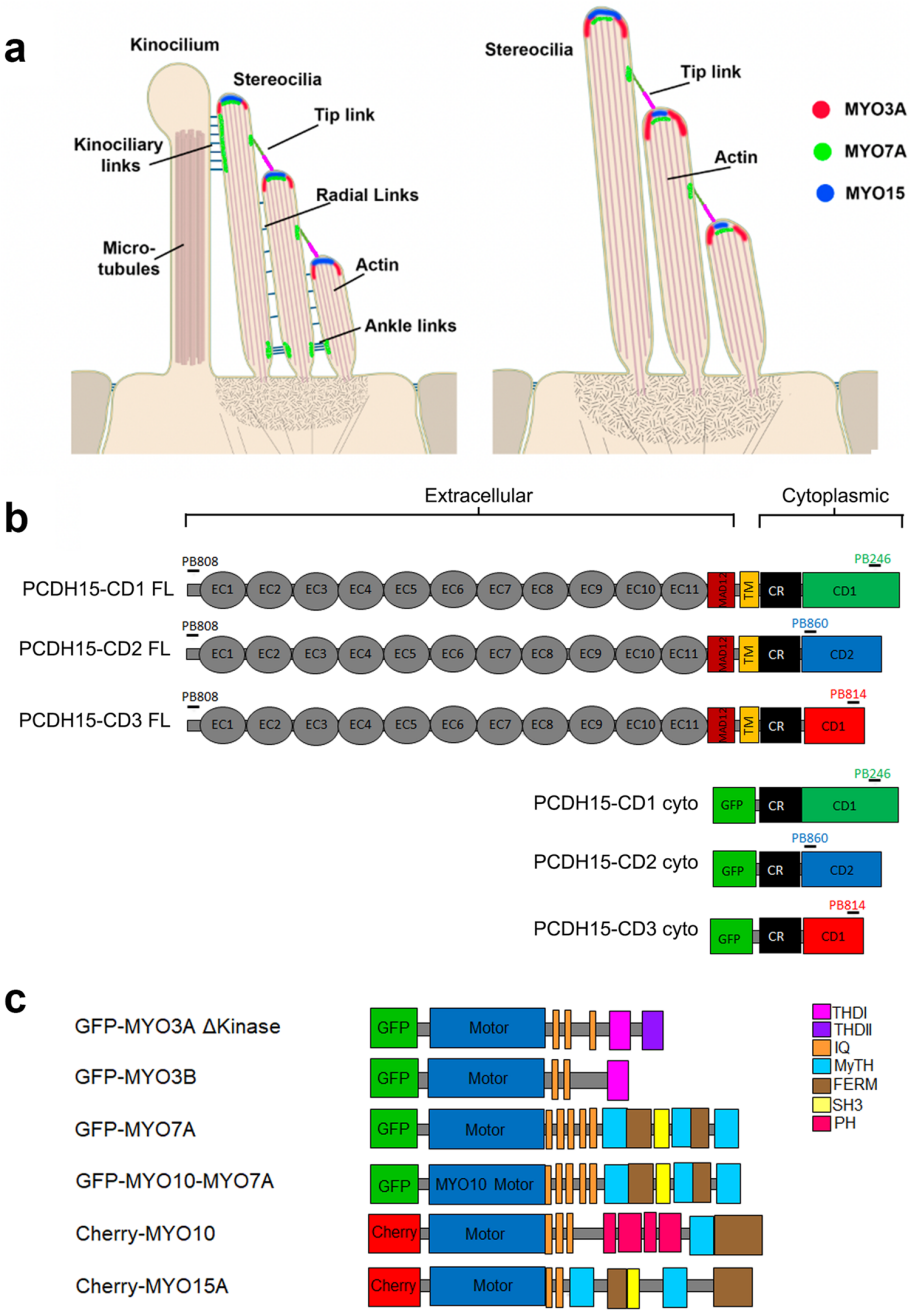
Localization of stereociliary myosin in the hair cell bundle and protein constructs used in this study. (**a**) Localization of MYO3A (red), MYO7A (green), and MYO15 (blue) in the hair cell bundle during development or in the vestibular organs (left panel) and in mature auditory hair cells (right panel). (**b**) Representation of the PCDH15 constructs used in this study. PCDH15 is a large cadherin protein with 11 extracellular calcium-binding (EC) domains, an extracellular linker or membrane adjacent domain (MAD12), a transmembrane^71^ segment, and a cytoplasmic domain. The cytoplasmic domain contains a common region (CR) and a unique tail domain specific to each isoform (CD1, CD2, and CD3). Constructs containing the cytoplasmic (cyto) domain of PCDH15-CD1, -CD2 and -CD3 were also generated. (**c**) Diagram of the myosin constructs used in this study.

During development of the stereocilia bundle of auditory hair cells, PCDH15 isoforms show a tipward localization, and are presumed to be part of the stereocilia tip links, radial links, and kinocilium-stereocilia links (Fig. 1a and Supplementary Fig. 1a). When the hair bundle matures, the radial links are pruned, the kinocilium is reabsorbed, and PCDH15 is mainly localized at the tips of the shorter stereocilia as part of the MET complex apparatus^26^. Vestibular hair cells follow a similar developmental transition, but their mature stereocilia bundles conserve the kinocilium (Fig. 1a and Supplementary Fig. 1b). The cargo-transporting stereocilia myosins 3A, 3B, 15A, and 7A also undergo changes in spatiotemporal localization during development and have been shown to dynamically localize at stereocilia tips (Fig. 1a) in mature bundles^7,18,20,31^. Of these myosins, MYO7A, presents a major localization change since it shows additional specialized accumulation near the stereocilia base and at the upper tip-link density in mature auditory hair cells^19,20,32^. To test for selective pairing of stereocilia myosins with PCDH15 isoforms as putative cargo, we examined their co-localization and dynamics along filopodia in cultured COS7 cells expressing combinations of these proteins.

### Generation and validation of PCDH15 constructs and antibodies

All three main PCDH15 isoforms; PCDH15-CD1, -CD2, and -CD3, which differ in their cytoplasmic domain (Fig. 1b), are expressed in the hair cell bundle^25,27^. We obtained or cloned murine full-length (FL) and generated cytoplasmic (cyto) domain cDNA constructs for all three isoforms. To track the PCDH15 isoform localization, we generated affinity purified isoform-specific antibodies targeting peptide sequences unique to each PCDH15 isoform, as well as a common peptide sequence from the conserved ectodomain region (Table 1). We confirmed the affinity and specificity of these antibodies in COS7 cells expressing the untagged PCDH15 FL isoforms (Supplementary Fig. 2) or their respective cyto-domains fused with a GFP at their N-termini (Supplementary Fig. 3d). All four antibodies showed selective labeling of PCDH15-expressing cells, and we did not detect any cross-reactivity of the isoform-specific antibodies with the other isoforms (Supplementary Figs. 2 and 3).

**Table 1:** Custom made polyclonal anti PCDH15 antibodies used in this study.

Antibody	Protein epitope	Epitope sequence
PB808	pan-PCDH15	GQYDDDWQYEDCKLARGG
PB246	PCDH15-CD1	PASNPQWGAEPHRHPK
PB860	PCDH15-CD2	EGEKARKNIVLARRRP
PB814	PDCD15-CD3	AVKPSGTRLKHTAE

The name, specific PCDH15 isoform recognized by these antibodies and the protein peptide used to generate the rabbit anti-PCDH15 antibodies are indicated.

The myosins used in this study were tagged at their N-termini with either GFP or mCherry (Fig. 1c). We used a MYO3A lacking its N-terminal regulatory kinase domain (MYO3A ΔKinase) since it localizes more efficiently to filopodia tips^33,34^. The short MYO15A isoform lacking the 133 kDa N-terminal domain was used to evaluate the transport of PCDH15 isoforms to filopodia tips. Because native MYO7A full length^35^ has an auto-inhibited state and does not induce filopodia in COS7 cells, we used a chimera that has a myosin 10 (MYO10) motor and a MYO7A tail domain that has been shown to induce filopodia formation and transport cargo to filopodia tips in COS7 cells^36^. To date, there is no evidence of MYO10 expression in hair cell stereocilia, but we included it in our experiments as a control for the MYO10 (motor)-MYO7A (tail) chimera (MYO10-MYO7A).

### MYO3A specifically and actively transports PCDH15-CD2 to the tips of filopodia

**Figure 2:**
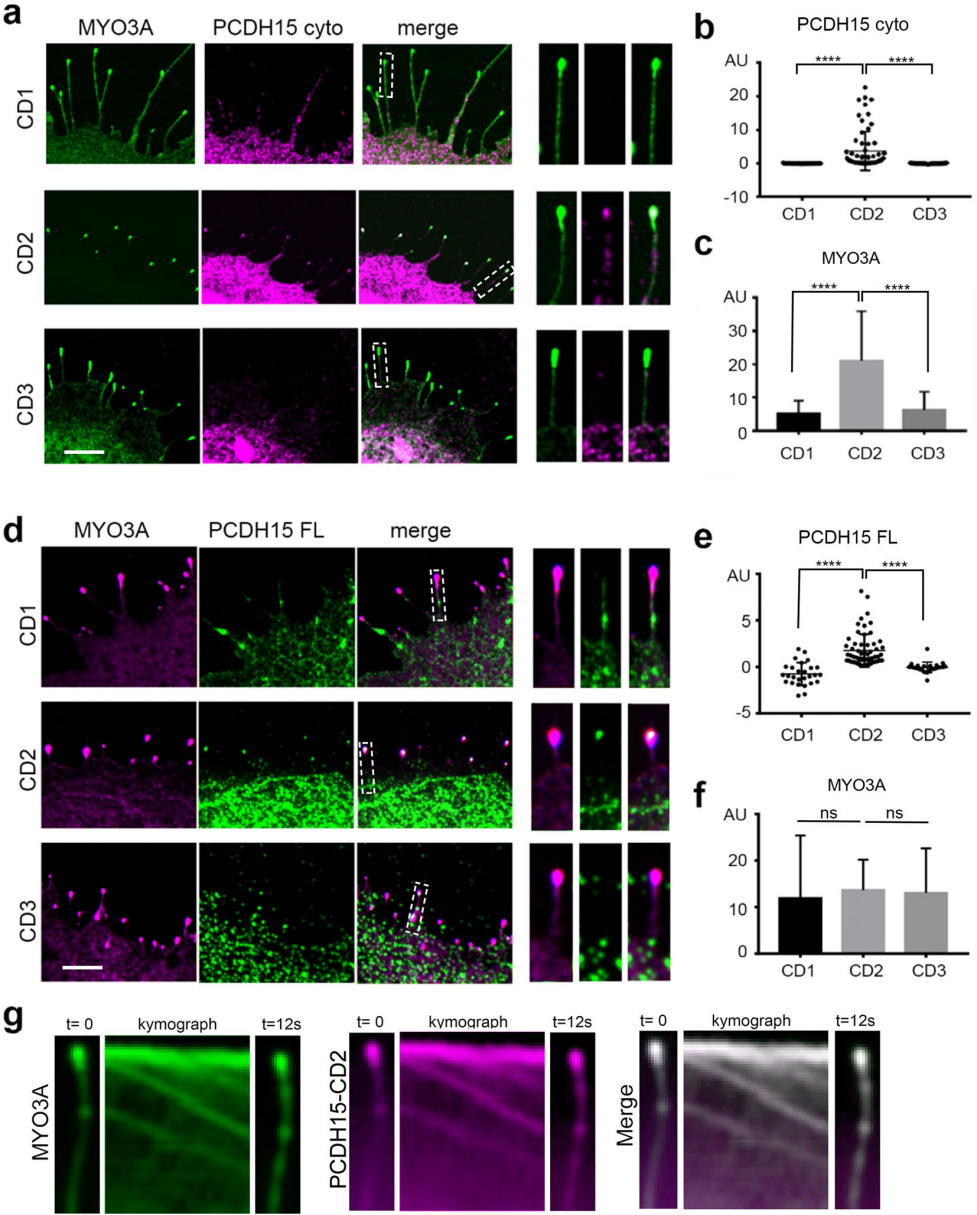
MYO3A actively transport PCDH15-CD2, but not CD1 or CD3, to the filopodia tips. (**a**) Confocal images of COS7 cells expressing GFP-MYO3A ΔK (green) and the cytoplasmic domain (cyto) of PCDH15-CD1, -CD2, or -CD3 (magenta). The right panels show a close-up view of a representative filopodia. (**b**) PCDH15-CD2 cyto exhibits significantly higher tip-to-shaft ratio than PCDH15-CD1 or PCDH15-CD3 when co-expressed with MYO3A. (**c**) Expression of PCDH15-CD2 enhanced the MYO3A enrichment at the filopodia tips (filopodia tip/shaft ratio). (**d**) Confocal images of COS7 cells expressing GFP-MYO3A ΔK (magenta) and Full-length PCDH15-CD1, -CD2, or -CD3 (green). (**e**) Full length PCDH15-CD2 exhibits significantly higher tip-to-shaft ratio than PCDH15-CD1 or PCDH15-CD3 when co-expressed with MYO3A. (**f**) MYO3A exhibit a similar enrichment at the filopodia tips when co-transfected with FL PCDH15-CD1, -CD2 or -CD3. Each dot in b and e represents one filopodia. Number of filopodia n = 23–66. One-way ANOVA analysis was performed with Dunnett’s multiple comparisons test. Level of significance was determined based on the p value (n.s p > 0.05, *p < 0.05, **p < 0.01, ***p < 0.001, ****p < 0.0001). (**g**) Kymographs depicting active transport of MYO3A and PCDH15-CD2 along a representative filopodium. Time elapsed from the first frame to the last was 12 s. The green and magenta fluorescence puncta correspond to clusters of MYO3A and PCDH15-CD2 co-translocating along the filipodium. Scale bars = 2 µm.

When MYO3A is expressed in COS7 cells it induces the formation of filopodia with MYO3A enriched at its tips, which provides a well-defined spatial compartment where potential interactions can be clearly visualized^18^. Cells co-expressing MYO3A and cyto PCDH15 showed that PCDH15-CD2, but not CD1 or CD3, is efficiently targeted to the tips of filopodia initiated by MYO3A (Fig. 2a). We quantified the distribution of PCDH15 by calculating the filopodia tip-to-shaft fluorescence ratio as described in the “Material and methods” section. We observed an increase in the filopodia tip-to-shaft ratio of PCDH15-CD2 while the expression of MYO3A did not produce any filopodia tip enrichment of either CD1 or CD3 isoforms (Fig. 2b). In addition, we quantified the enrichment of MYO3A at the filopodia tips by examining the filopodia tip-to-shaft fluorescence ratio. Interestingly, we observed that that the enrichment of MYO3A at the filopodia tips was significantly enhanced in the presence of the cyto PCDH15-CD2 but not in the presence of-CD1 and-CD3 isoforms (Fig. 2c), suggesting some form of cooperativity between the MYO3A motor activity and the PCDH15-CD2 cargo.

To confirm that the FL PCDH15-CD2 isoforms can also be transported by MYO3A to filopodia tips, we performed similar experiments with FL PCDH15-CD1, -CD2 and -CD3 (Fig. 2d–f). We observed that FL PDH15-CD2, but not -CD1 or -CD3, was enriched at the tips of MYO3A filopodia (Fig. 2e). In fact, we observed a higher tip-to-shaft ratio of FL PCDH15-CD2 when compared to cyto PCDH15-CD2, while the expression of MYO3A did not alter the cellular localization of the -CD1 or -CD3 isoforms. However, the increase in the MYO3A filopodia tip-to-shaft ratio with FL PCDH15-CD2 (Fig. 2f) was not as striking as with the cyto PCDH15-CD2 and MYO3A. Additionally, live-cell imaging in COS7 cell expressing fluorescently tagged MYO3A and cyto-PCDH15-CD2 shows that the localization and enrichment of PCDH15-CD2 at filopodia tips is the result of active transport by MYO3A. Filopodia of COS7 cells transfected with GFP-MYO3A and mCherry-cyto-CD2 showed dynamic localization of both proteins at filopodia tips from the early steps of their initiation and elongation. Kymographs of the filopodia also showed that the two proteins leave the filopodia tips and co-translocate towards the filopodia base (Fig. 2g). This retrograde motion is due to clusters of the myosin and its cargo riding the rearward treadmilling of the actin that makes up the filopodia core^37^, indicating that MYO3A and PCDH15-CD2 maintain interaction as they translocate along actin.

### ESPN1 and ESPNL differentially regulate the transport of PCDH15-CD2 by class III myosins

**Figure 3:**
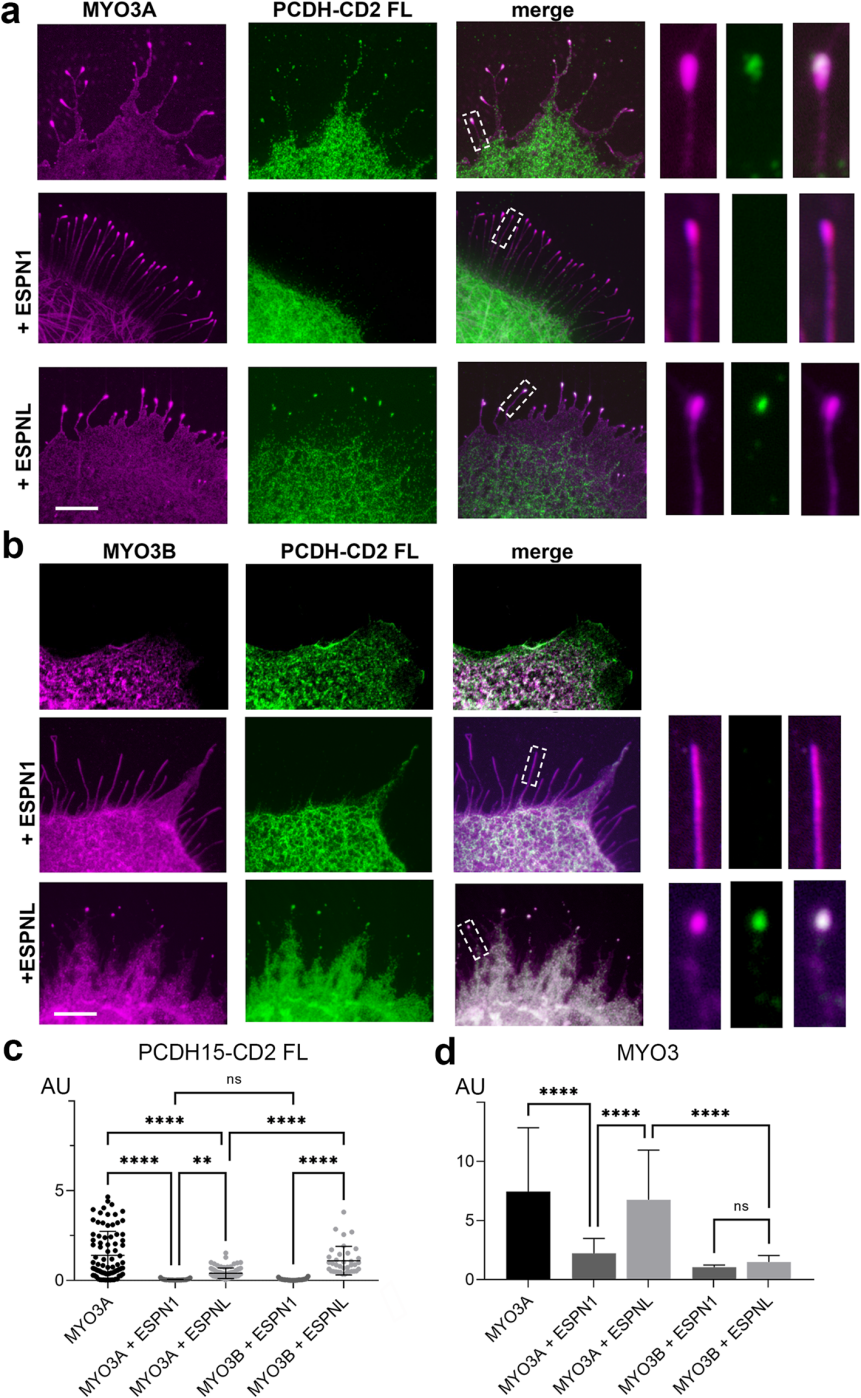
ESPN1 and ESPNL differentially regulate the transport of PCDH15-CD2 by class III myosins. (**a**) Confocal images of COS7 cells expressing GFP-MYO3A (magenta) and PCDH15-CD2 FL (green) in the absence or presence of ESPN1 (espin-1) or ESPNL (espin-like). (**b**) Confocal images of COS7 cells expressing GFP-MYO3B (magenta), PCDH15-CD2 FL (green) in the absence or presence of ESPN1 or ESPNL. Filopodia zoom in images are shown at the right. (**c**) PCDH15-CD2 enrichment at the filopodia tips in the conditions shown in (**a**) and (**b**). Mean ± SD is shown. Each dot represents one filopodia. Number of filopodia n = 28–97. (**d**) MYO3A relative enrichment at filopodia tips for each of the conditions examined. One-way ANOVA analysis was performed with Dunnett’s multiple comparisons test. Level of significance was determined based on the p value (n.s p > 0.05, *p < 0.05, **p < 0.01, ***p < 0.001, ****p < 0.0001). Scale bar = 2 µm.

The class III myosin cargoes espin-1 (ESPN1) or espin-like (ESPNL) bind to the tail homology domain I (THDI) of MYO3A and MYO3B to differentially regulate molecular transport and control filopodia growth and are essential for normal hearing^29^. Therefore, we next examined whether expression of ESPN1 and ESPNL alters the transport of PCDH15-CD2 FL to the filopodia tips. Cells expressing MYO3A, PCDH15-CD2 and ESPN1 presented the characteristic long filopodia of cells expressing a class III myosin and ESPN1^48^. However, while cells expressing MYO3A and PCDH15-CD2 FL have these two proteins enriched at their filopodia tips, PCDH15-CD2 FL is no longer enriched at the filopodia tips in cells expressing MYO3A and ESPN1 (Fig. 3a,c), suggesting that ESPN1 prevents the transport of PCDH15-CD2 by MYO3A. We next analyzed the transport of PCDH15-CD2 by MYO3A in the presence of the ESPN1 paralog, ESPNL. Importantly, we focus our study on cells expressing low levels of ESPNL, since high expression levels inhibit MYO3A-dependent filopodia formation^29^. In contrast to that observed with ESPN1, PCDH15-CD2 FL was enriched at the filopodia tips of COS7 cells expressing MYO3A and ESPNL (Fig. 3a) although PCDH15-CD2 FL was less enriched at the MYO3A filopodia tips in the presence of ESPNL than in the absence (Fig. 3c), suggesting that MYO3A can transport ESPNL and PCDH15-CD2 simultaneously. We observed an increased number in filopodia per 10 mm in cell expressing MYO3A, CD2 and ESPNL (3.96 ± 1.01) when compared to cells expressing MYO3A and CD2 (2.43 ± 0.69), like previously reported for cells expressing MYO3A and ESPNL^29^, suggesting that ESPNL is transported to the filopodia tips. Quantification of MYO3A enrichment at the filopodia tips in absence or presence of ESPNL revealed that ESPNL did not affect MYO3A enrichment (Fig. 3d). These data indicate that the presence of ESPNL does not affect the transport of PCDH15-CD2 by MYO3A and reveal an intricate regulatory role for MYO3A cargos in the transport of PCDH15-CD2.

The shorter myosin class III isoform, MYO3B, lacks the additional actin-binding domain that MYO3A presents on its tail (3THDII) (Fig. 1c), which prevents this myosin from translocating along actin and concentrating at the tips of filopodia by itself^7,38^ (Fig. 3b). Consequently, to move along the actin filaments and reach the tips of stereocilia or filopodia, MYO3B requires the actin-binding activity of ESPN1^7^ or ESPNL^29^. We thus examined whether MYO3B transports PCDH15-CD2 FL to the filopodia tips when co-expressed with ESPN1 or ESPNL in COS7 cells (Fig. 3b–d). Like previously reported in cells expressing MYO3 and ESPN1 or ESPNL in the absence of PCDH15-CD2^7,29^, we observed that MYO3B enrichment at the filopodia tips is less efficient than MYO3A, and that MYO3B was enriched at the filopodia tips in the presence of ESPN1 or ESPNL (Fig. 3d). These data suggest that expression of PCDH15-CD2 doesn’t alter MYO3A nor MYO3B trafficking to the filopodia tips. However, like that observed with MYO3A, we found that PCDH15-CD2 FL was enriched at the filopodia tips of cells expressing MYO3B and ESPNL but not ESPN1 (Fig. 3b,c), suggesting that ESPN1 and ESPNL differentially regulate the transport of PCDH15-CD2 by MYO3A and MYO3B.

### MYO7A transports PCDH15-CD3 to the tips of filopodia

Immunoprecipitation experiments have shown that the tail domain of MYO7A interacts with the cytoplasmic domain of PCDH15 cloned from mouse brain tissue^32^, suggesting that these proteins could cooperate to regulate hair cell bundle development and function. Contrary to the other myosins tested in this study, class VII myosins maintain a cytoplasmic non-motile conformation and do not form filopodia by themselves^35^. Therefore, to test the role of MYO7A in the transport of the three PCDH15 isoforms, we used a chimeric MYO10-MYO7A myosin containing the motor domain of MYO10 and the tail cargo-binding domain of MYO7A (Fig. 1c), which is sufficient to translocate on actin and induce filopodia formation^35,39,40^.

**Figure 4:**
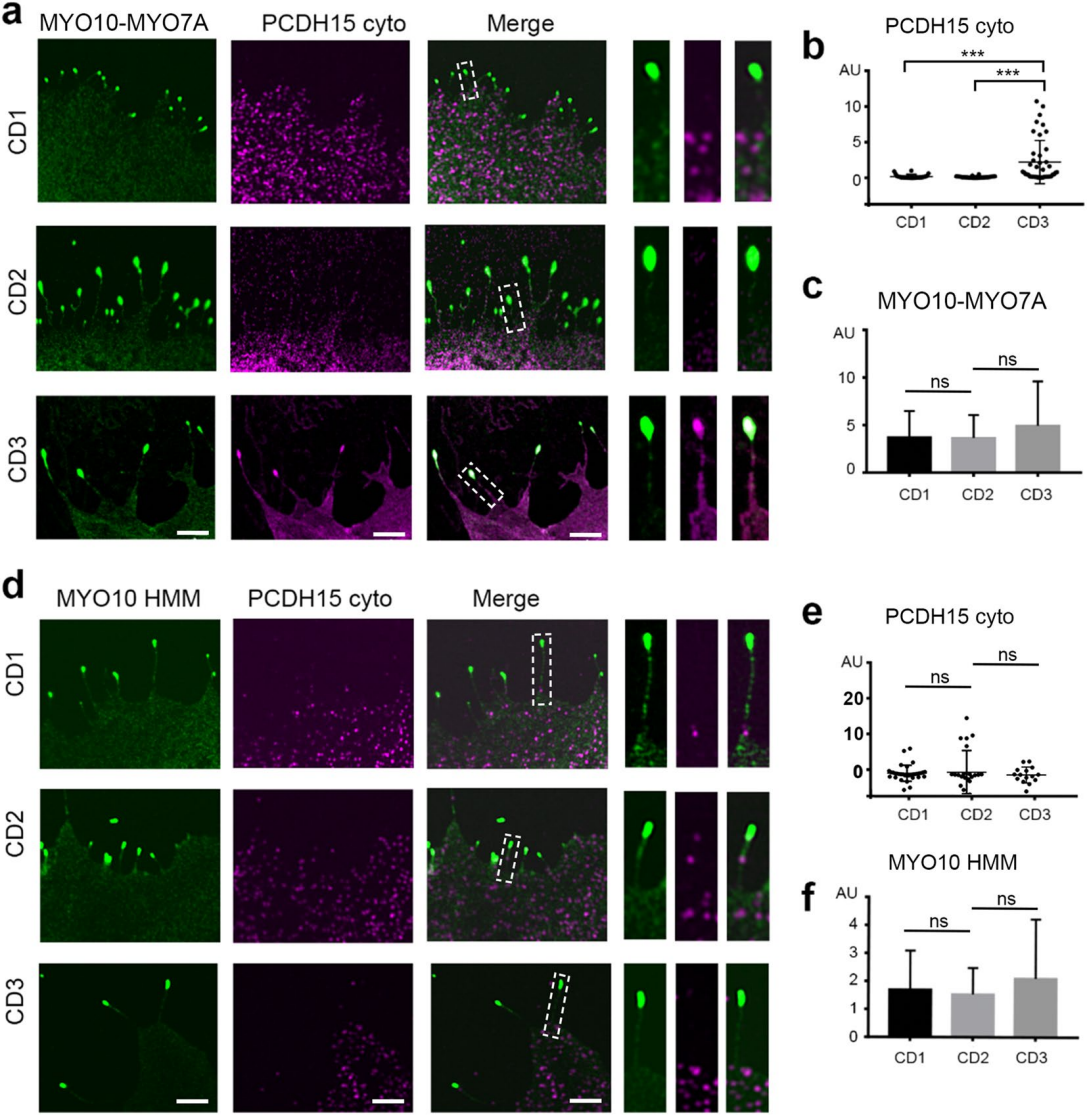
MYO7A tail enables transport of PCDH15-CD3 to the tips of filopodia. (**a**) Confocal images of COS7 cells expressing the chimeric MYO10-MYO7A protein (green) and cyto PCDH15-CD1, -CD2 or -CD3 (magenta). The right panels show a close-up view of a representative filopodia. (**b**) PCDH15-CD3 cyto exhibits significantly higher tip-to-shaft ratio than PCDH15-CD1 or PCDH15-CD2 when co-expressed with MYO10-MYO7A. (**c**) MYO10-MYO7A exhibit a similar enrichment at the filopodia tips when co-transfected with FL PCDH15-CD1, -CD2 or -CD3. (**d**) Confocal images of COS7 cells expressing the MYO10 (green) and cyto PCDH15-CD1, -CD2 or -CD3 (magenta). The right panels show a close-up view of a representative filopodia. (**e**) We did not observe a significantly change in the tip-to-shaft ratio of any of the PCDH5 isoform tested. (**f**) MYO10 HMM exhibited a similar enrichment at the filopodia tips when co-transfected with FL PCDH15-CD1, -CD2 or -CD3. Each dot in b and e represents one filopodia. Number of filopodia n = 15–43. One-way ANOVA analysis was performed with Dunnett’s multiple comparisons test. Level of significance was determined based on the p value (n.s p > 0.05, *p < 0.05, **p < 0.01, ***p < 0.001, ****p < 0.0001). Scale bar = 2 µm.

Cell expressing MYO10-MYO7A and PCDH15-CD3 show robust co-enrichment of the two proteins at the tips of filopodia (Fig. 4a,b), suggesting that MYO7A tail specifically transports PCDH15-CD3. However, we did not observe enrichment of PCDH15-CD1 nor PCDH15-CD2 at the filopodia tips of cells expressing MYO10-MYO7A. Furthermore, we did not observe any difference in the enrichment of MYO10-MYO7A at the tips of filopodia when expressed in combination with PCDH15-CD1, -CD2, or -CD3 (Fig. 4c), suggesting that the PCDH15-CD3 cargo is not influencing MYO10-MYO7A motor activity. To confirm that PCDH15-CD3 transport is dependent on the MYO7A tail, and not the MYO10 portion of the chimeric MYO10-MYO7A, we performed similar experiments using MYO10 heavy meromyosin (HMM), consisting of the head, neck, and α-helical region of MYO10. While MYO10 was equally enriched at the tips of filopodia of COS7 cell it was not accompanied with tip enrichment of any of the PCDH15 isoforms, including CD3 (Fig. 4d,e). These data suggest that the MYO7A tail is required for the transport of PCDH15-CD3.

Interestingly, MYO7A cargoes have been shown to promote the dimerization and filopodia formation activity of MYO7A^40^. Since our data suggest that PCDH15-CD3 might be a MYO7A cargo, we examined whether PCDH15-CD3 can promote filopodia formation by wild type MYO7A. COS7 cells expressing PCDH15-CD3 and MYO7A showed a diffuse intracellular localization of MYO7A and do not present filopodia, indicating that PCDH15-CD3 does not activate MYO7A to move along actin structures as a cargo transporting motor (Supplementary Fig. 4).

### MYO15A, a principal cargo transport in stereocilia, does not transport PCDH15 to the tips of filopodia

**Figure 5:**
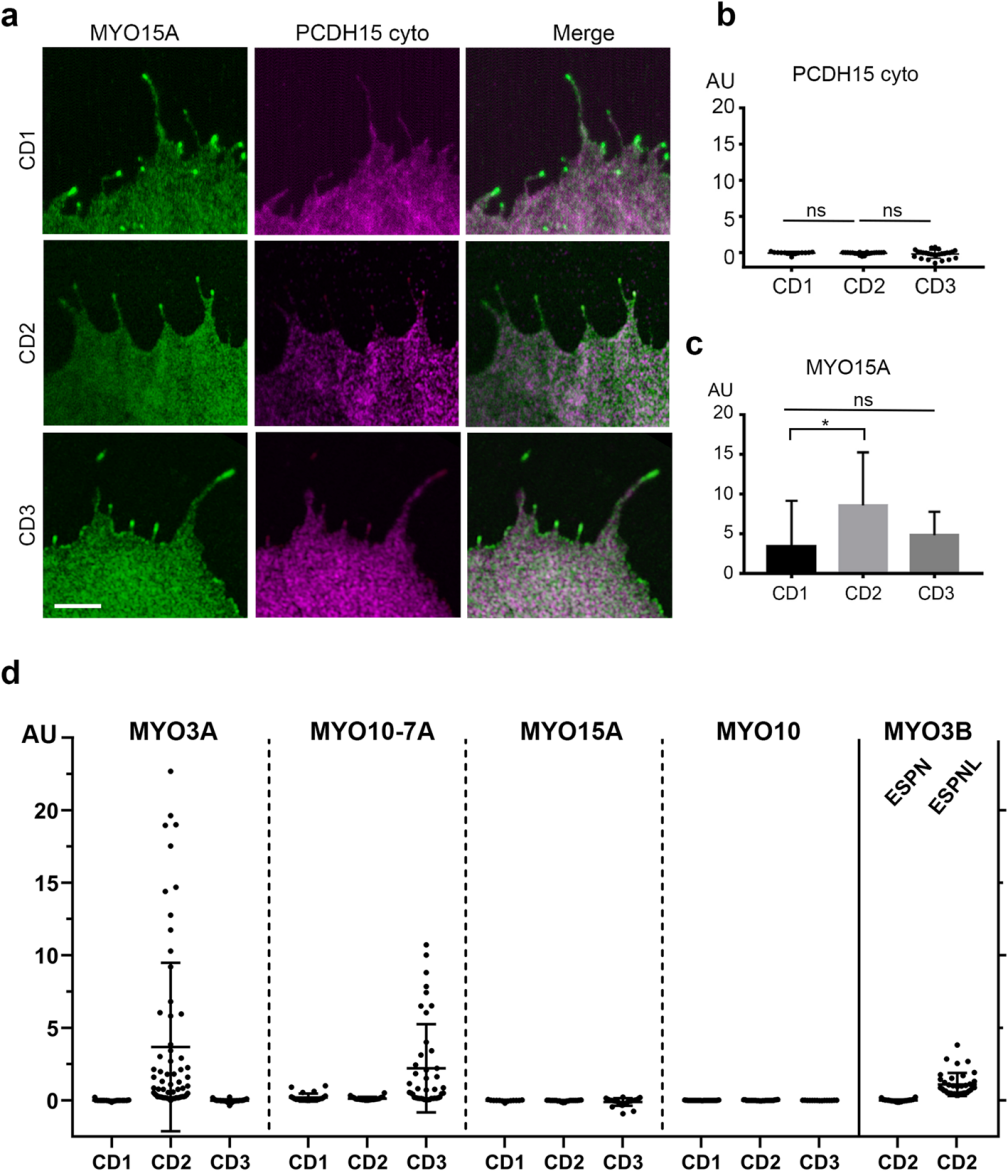
MYO15A does not transport PCDH15 to the tips of filopodia. (**a**) Confocal images of COS7 cells expressing MYO15A (green) and cyto PCDH15-CD1, -CD2 or -CD3 (magenta). (**b**) None of the PCDH15 isoforms showed a significantly tip-to-shaft ratio when co-expressed with MYO15A. (**c**) MYO15A exhibit a slightly higher enrichment at the filopodia tips when co-transfected with cyto PCDH15-CD2 when compared to -CD1 or -CD3. (**d**) Summary of the tip-to-shaft ratio of PCDH15 isoforms when expressed with MYO3A, MYO10-MYO7A, MYO15A, MYO10 and MYO3B in the presence of ESPN1 or ESPNL. Each dot in b and d represents one filopodia. Number of filopodia n = 13–66. One-way ANOVA analysis was performed with Dunnett’s multiple comparisons test. Level of significance was determined based on the p value (n.s p > 0.05, *p < 0.05, **p < 0.01, ***p < 0.001, ****p < 0.0001). Scale bar = 2 µm.

MYO15A transports GPSM2, GNAI3, WHRN and EPS8 to the stereocilia tips and mutation in these proteins results in shortening of stereocilia and hearing loss^8,41–44^. Using the same co-expression assay, we tested the potential role of the short isoform of MYO15A in the transport of PCDH15 isoforms. We observed that while MYO15A induces filopodia formation and is enriched at the filopodia tips of COS7 cells (Fig. 5a), it did not promote the enrichment of PCDH15-CD1, -CD2 nor -CD3 at the filopodia tips (Fig. 5b). Therefore, our data does not support a direct role for the short isoform of MYO15A^45^ in the transport of any of the three main PCDH15 isoforms in stereocilia.

## Discussion

The mechanisms of transport and assembly of molecular components of the MET apparatus at stereocilia tips remains a key question in hair cell biology. The characterization of direct interactions and individual roles for each molecular MET component has been challenging due to the molecular crowding at stereocilia tips, the mutual influence that each protein can exert on each other, and the compensatory roles reported for several molecular components. In this work, we took a heterologous expression approach to dissect the role myosins commonly detected in stereocilia may have in the selective transport of PCDH15 isoforms in filopodia. Filopodia and stereocilia are actin-based protrusions that emerge from the cytoskeleton through the action of actin regulatory proteins that initiate, elongate, and precisely regulate the architecture and length of these cellular protrusions. Myosins transport cargoes along the length of these protrusions, enriching the tips of filopodia and stereocilia tips with adhesion molecules, actin-regulatory proteins and other factors that are essential for their formation and function. While specific proteins differentiate both protrusions^46,47^ and recent data suggest that stereocilia present an unique actin turnover mechanism different from that observed in the more dynamic filopodia^5,21,48–50^, filopodia formation in COS7 cells is a valuable model system for understanding how myosins transport cargoes along crosslinked parallel actin protrusions, such hair cell stereocilia. With this approach, we demonstrate that MYO3A selectively transports PCDH15-CD2 (Fig. 2) while MYO7A transports PCDH15-CD3 (Fig. 4). Surprisingly, the short isoform of MYO15A, which is another key stereocilia cargo transport myosin and localizes at stereocilia tips did not show interactions with any of the three PCDH15 isoforms (Fig. 5). Figure 5d summarize our tip-to-shaft PCDH15 enrichment data with each specific myosin motor. The two MYO15A isoforms recently identified in hair cell stereocilia differ in the presence of a N-terminal 133 kDa domain but share the cargo binding domains in their C-terminal tail^45^. Therefore, while cannot rule out the role of the MYO15A N-terminal domain in PCDH15 transport, the fact that MYO15A isoforms share the cargo binding domain suggest a preserved cargo transport function for both isoforms. Furthermore, our results indicating a minor role of MYO15A in PCDH15 transport are consistent with the reported preservation of MET in the shaker-2 mouse model lacking the long and short isoforms of MYO15A^51^.

Interestingly, none of the myosins tested showed effective transport of the PCDH15-CD1 isoform. Senften and colleagues^32^ reported that PCDH15 from mouse brain binds to the SRC Homology 3 (SH3) domain of MYO7A and recruits MYO7A to the cell membrane in cultured cells. While the specific PCDH15 isoform used in this study was not specified, its sequence length corresponds to the CD1 isoform, suggesting that MYO7A interacts with PCDH15-CD1. However, we observed that MYO7A transports PCDH15-CD3, but not PCDH15-CD1, to the filopodia tips. Therefore, it is possible that while several PCDH15 isoforms might interact with MYO7A, other factors or adaptor proteins may be required for the transport of PCDH15-CD1 by MYO7A at the filopodia tips. Consistent with this, hair cells from shaker1 mice lacking MYO7A failed to localize PCDH15-CD1 at the stereocilia tips^32^. Likewise, cargo or adaptor-dependent regulation of myosin transport has been established in the literature^52^ and is also evident in our experiments where ESPN1 interfered with the MYO3A-dependent enrichment of PCDH15-CD2 at filopodia tips (Fig. 3).

Myosins and espins are directly involved in regulation of actin filament formation and stability including in the differential regulation of stereocilia length. ESPN1 and ESPNL bind to the THDI of MYO3A and MYO3B and can mutually influence myosin activity and actin polymerization^30^. Differential expression of these proteins correlates with differential lengths of stereocilia in each bundle and across different hair cells along the organ of Corti or in the different inner ear sensory epithelia^29,53^. The dynamic localization of PCDH15-CD2 at filopodia tips when co-transfected with MYO3A indicate that MYO3A specifically interacts with and transports PCDH15-CD2. PCDH15-CD2 transport by MYO3A and MYO3B in the presence of ESPNL suggest that PCDH15-CD2 may interact with a common domain in these class III myosins. nhibition of PCDH15-CD2 transport by ESPN1 suggest that these two MYO3A cargoes could compete for a common MYO3A binding site. However, in this scenario, we would have expected ESPNL to also inhibit PCDH15-CD2 transport since ESPN1 and ESPNL bind to the THD1 domain of MYO3A and MYO3B in a conserved way^54^. Therefore, a more complex mechanism may be in place by which ESPN1 and ESPNL can differentially regulate PCDH15-CD2 transport and enrichment at the tips of actin protrusions. ESPN1 and ESPNL are known to influence the length and structure of filopodia and stereocilia^7,29,53,55^. It is also likely that the transport and enrichment of cargoes depend on the length and number of actin tracks in an actin protrusion. Consequently, the transport of actin regulatory proteins by MYO3A and 3B may influence the transport of PCDH15-CD2 in multiple ways and modulate the relationship between stereocilia length and MET. Remarkably, the localization of MYO3 cargoes, ESPNL, ESPN1, MORN4 and PCDH15, is preserved at the stereocilia tips of hair cells from Myo3a^−/−^; Myo3b^−/−^ double knock-out mice^56^, suggesting the presence of a compensatory mechanism for the transport of MYO3 cargoes and highlighting the complexity of the protein transport mechanisms in hair cells.

Our results highlight the intricate nature of the mechanisms of molecular cargo transport and assembly of the MET complex that takes place during development, maturation, life-long function, and likely in the degeneration of the stereocilia bundles during aging. Several isoforms of PCDH15 differing in their cytoplasmic domains are expressed in the hair cell bundle, suggesting that alternative splicing regulates PCDH15 localization and function. The main three PCDH15-CD1, PCDH15-CD2 and PCDH15-CD3 isoforms can compensate for each other in the radial-link formation and the tip link formation of immature hair cells^25,26^ (Fig. 1a). However, PCDH15-CD2 has an essential role in the formation of the tip link in mature hair cells. Another essential component of the MET channel complex is the membrane protein TMIE, which has been shown to specifically interact with PCDH15-CD2^28,57^. Interestingly, appearance and expression levels of MYO3A at stereocilia tips during development are correlated with onset and maturation of MET^58^. Taken together these data suggest that MYO3A and MYO3B play a role in the assembly and maturation of the MET complex. Given that MYO3A and MYO3B are intra- and inter-molecularly regulated by their kinase domain and the presence of several calmodulin binding sites, they can also potentially exhibit Ca^2+^-dependent regulation^59–61^. Therefore, we cannot rule out their participation in the MET adaption processes. Consistent with this, a recent report provides good evidence that MET adaptation takes place at or near the channel complex^62^.

While PCDH15 has not been shown to directly affect actin regulation, its proper targeting to the stereocilia tip and participation in the tip link and MET is required for normal length regulation of stereocilia as it has been shown in various experimental conditions and mouse models^3–5^. Furthermore, the selective transport of PCDH15-CD2 and PCDH15-CD3 by MYO3A and MYO7A could regulate the composition and properties of the MET channel influencing the transport of other essential MET complex components know to interact with PCDH15^57,63,64^. Interestingly, all the myosins and the cargoes considered in this study have been directly involved in multiple syndromic and non-syndromic forms of hearing loss and vestibular dysfunction^30,41,65–69^. Our results showing the intricate relationships between these molecular components provide new insights into potential common molecular and structural mechanisms underlying loss of function.

Our observations in the reductionist heterologous expression system allowed us to identify specific interactions that would otherwise be concealed in the complex and interconnected context of stereocilia bundles in mouse models. However, additional work is necessary to elucidate how PCDH15-CD1 and other component of the MET channel complex are transported, and how the myosin and cargo systems are regulated and the extent of their cooperativity and complementarity in normal and affected hair cells during development, homeostasis, and aging.

## Material and methods

### Expression plasmids

The full length of human PCDH15-CD1-1 (Q99PJ1, uniport) cloned in pcDNA3.1 was obtained from OriGene. PCDH15-CD2 (Q99PJ1-10, uniport) and PCDH15-CD3-1 (Q99PJ1-18, uniport) were cloned from genomic mouse DNA. The cytoplasmic domains of the PCDH15 isoforms corresponding to residues 1403–1443 of PCDH15-CD1, 1410–1790 of PCDH15-CD2, and 1403–1682 of PCH15-CD3, were PCR amplified from the full-length cDNAs and cloned into the NheI and BamHI or into the HindIII and BamHI restriction sites of the pEGFP-C2 vectors (Clontech), resulting in untagged soluble proteins or proteins tagged with a GFP fused to its N-terminus, respectively. For live imaging experiments, the PCDH15-CD2 isoform cytoplasmic domain was cloned using the HindIII and BamHI restriction sites of the pmCherry-C2 vector (Clontech), resulting in soluble protein tagged with cherry at its N-terminus.

The human MYO3A and mouse MYO3B constructs used in our study lack the N-terminal kinase domain of class III myosins (MYO3A-ΔK and MYO3B-ΔK) and thus localize more efficiently to the stereocilia tips than the full-length MYO3A protein. The kinase domain has been shown to downregulate motor activity leading to a slower MYO3A protein^33^. The ESPN1, ESPNL, GFP-MYO3A, GFP-MYO3B, and mCherry-MYO3B cDNAs were previously prepared and used in our laboratory and described^7,29,53^. The MYO15A used in our experiments corresponds to the isoform 2 lacking the N-terminal 133 kDa domain.

### Antibodies

Affinity purified rabbit polyclonal anti-mouse PCDH15 antibody (COVANCE) generated against the N-terminus domain of PCDH15 conserved in all the PCDH15 isoform was used to detect all the PCDH15 isoforms (pan-PCDH15 antibody, PB808). Affinity purified rabbit polyclonal anti-mouse PCDH15 antibodies (COVANCE) generated against the variable intracellular cytoplasmic domain (CD) was used to specifically detect the PCDH15 isoforms PCDH15-CD1, PCDH15-CD2, and PCDH15-CD3 (PB246, PB860, and PB814, respectively) (Table 1). The four custom-made antibodies used in this manuscript were validated in transfected COS7 cells expressing the three PCDH15 isoforms as shown in the Supplementary Fig. 2 and explained in the following confocal microscopy analysis section.

### Confocal microscopy imaging

COS7 cells (ATCC, CRL-1651) were grown and maintained in Dulbecco’s Modified Eagle Medium (DMEM) supplemented with 10% fetal bovine serum (FBS) at 37 °C and 5% CO_2_ in a cell incubator. Cells were trypsinized and plated on glass coverslips at a 50% confluency, and 18 h later, they were transfected with 1 μg of cDNA using the Lipofectamine transfection reagent (Invitrogen) per manufacturer’s instructions. Cell media was changed 18 h after transfection to remove the residual Lipofectamine and cells were maintained in the cell incubator during 24 h for protein expression. Samples were then fixed for 20 min in 2% paraformaldehyde in Phosphate Buffered Saline (PBS), permeabilized and counterstained for actin with a 1:100 dilution of CF-405 M Phalloidin (Biotium) in 0.5% Triton X-100 in PBS for 30 min. After removing TritonX-100 and phalloidin with 2–3 PBS washes, samples were mounted on glass slides and imaged in a Nikon microscope equipped with a Yokogawa spinning disk confocal unit.

When expressing the untagged PCDH15 isoforms, cells were permeabilized 0.5% Triton X-100 in PBS for 30 min and the excess Triton X-100 was were removed with 2–3 PBS washes, and 4% Bovine Serum Albumin (BSA) in PBS was added to the cells and incubated for 1 h. Primary rabbit polyclonal antibodies against PCHD15 (PB808, PB246, PB860, or PB814) at a 1:500 dilution in PBS with 4% BSA was added and incubated with the cells for 1 h at RT. After 2–3 washes with PBS to remove unbound antibody, secondary Alexa flour 564 or Alexa flour 643 anti-rabbit (Life Technology) at a 1:2000 dilution in PBS with 4% BSA was added and incubated for 30 min together with CF405M-Phalloidin (Biotium) at a 1:100 dilution to label F-actin. Cells were then washed with PBS several times and mounted using Celvol 205 mounting media in microscopy slides for confocal imaging. Imaging was performed in a Nikon microscope equipped with a Yokogawa spinning disk confocal unit.

### Quantification of fluorescently tagged and immunolabeled proteins along the filopodia

Microscopy data processing, analysis, and estimation of the relative pixel intensity of fluorescently tagged proteins along the filopodia were done in ImageJ^70^. To quantify the enrichment at the filopodia tips, we measured the filopodia tip-to-shaft fluorescence intensity ratio. To do this, we used the line tool from ImageJ to draw three lines of one pixel in width; one along the filopodia tip, other one along the filipodia shaft, and a third one in a region outside filopodia. The line drawn near but outside the filopodia was considered as background and subtracted from the fluorescence measurements. We calculated the ratio of tip vs shaft or estimated the enrichment at the filopodia tips by subtracting the fluorescent intensity at the filopodia shaft from the one at the tip. To measure the enrichment at the filopodia, we measure the filopodia tips to cell fluorescence intensity ratio. All experiments were performed three times and quantification was done in 15–97 filopodia from 3 to 5 cells. The data was imported into the GraphPad Prism 8 software for the generation of the graphs and statistical analysis.

### Immunolabeling of mammalian hair cells

All mice used in this work were handled following the NIH Guidelines for Animal Care and approved by the NIDCD Animal Protocol Committee (Protocol #1215), and all animals received humane care in accordance with the ARRIVE guidelines developed by the National Centre for the Replacement, Refinement, and Reduction of Animals in Research. Cochlear tissue from wild type 10 days old mice or vestibular tissue from adult guinea pig was rapidly isolated in Leibovitz L-15 medium and fixed in 4% formaldehyde in HBSS buffer for 20 min and then washed with phosphate-buffered saline (PBS). Samples were then permeabilized with 0.5% Triton X-100 in PBS for 20 min before blocking with bovine serum albumin (BSA). After blocking for 1 h with 1% BSA, 3% normal goat serum in PBS, organs were incubated overnight at 4 °C with primary antibodies in blocking buffer. Custom-made pan PCDH5 antibody PB811 or a mixture of the pan PCDH15 containing PB811 and PB808 antibodies were used to detect all the PCDH15 isoforms. Organs were then washed 3 × 5 min with PBS and incubated with secondary antibodies (Alexa 546-conjugated goat anti-rabbit IgG) containing Alexa Fluor 405 phalloidin (to label stereocilia actin) for 30 min, before washing five times in PBS and mounting between a microscope slide and cover slip. Samples were viewed in a Nikon inverted fluorescence microscope, fitted with a spinning disk confocal scan head (Yokogawa, CSU-22), 100 × Apo 1.49 numerical aperture objective, and an EM-CCD (Andor 888 or 897) camera. Nikon NIS-Elements imaging software was utilized for image acquisition and analysis. This study is reported in accordance with ARRIVE guidelines.

## Supplementary Information

Supplementary Figures.

## Data Availability

The datasets generated and/or analyzed during the current study are available from the corresponding author on reasonable request.
