# Supplementary material for: Selective binding and transport of protocadherin 15 isoforms by stereocilia unconventional myosins in a heterologous expression system
Source: Sci Rep. 2022 Aug 12;12:13764. doi: 10.1038/s41598-022-17757-0 (PMC9374675; doi:10.1038/s41598-022-17757-0)
Supplement: Supplementary file 1 — Supplementary Figures. [file 41598_2022_17757_MOESM1_ESM.docx]

**SUPPEMENTARY MATERIAL**

**Selective binding and transport of protocadherin 15 isoforms by stereocilia unconventional myosins in a heterologous expression system**

Angela Ballesteros ^Ψ 1 *^, Manoj Yadav ^Ψ 1^, Runjia Cui^1^, Kiyoto Kurima^2^, and Bechara Kachar^1*^

^1^Laboratory of Cell Structure and Dynamics, National Institute on Deafness and Other Communication Disorders, National Institutes of Health, Bethesda, MD, USA.

^2^Molecular Biology and Genetics Section, National Institute on Deafness and other Communication Disorders, National Institutes of Health, Bethesda, MD, USA.

^Ψ^ These authors contributed equally.

*Corresponding authors:

Angela Ballesteros, Ph.D.

Current address: Molecular Physiology and Biophysics Section, NINDS, NIH, Bethesda, MD 20892. Email: [angela.ballesteros@nih.gov](mailto:angela.ballesteros@nih.gov)

and

Bechara Kachar, M.D.

Laboratory of Cell Structure and Dynamics, NIDCD, NIH, Bethesda, MD 20892, USA.

Email: [kacharb@nidcd.nih.gov](mailto:kacharb@nidcd.nih.gov)


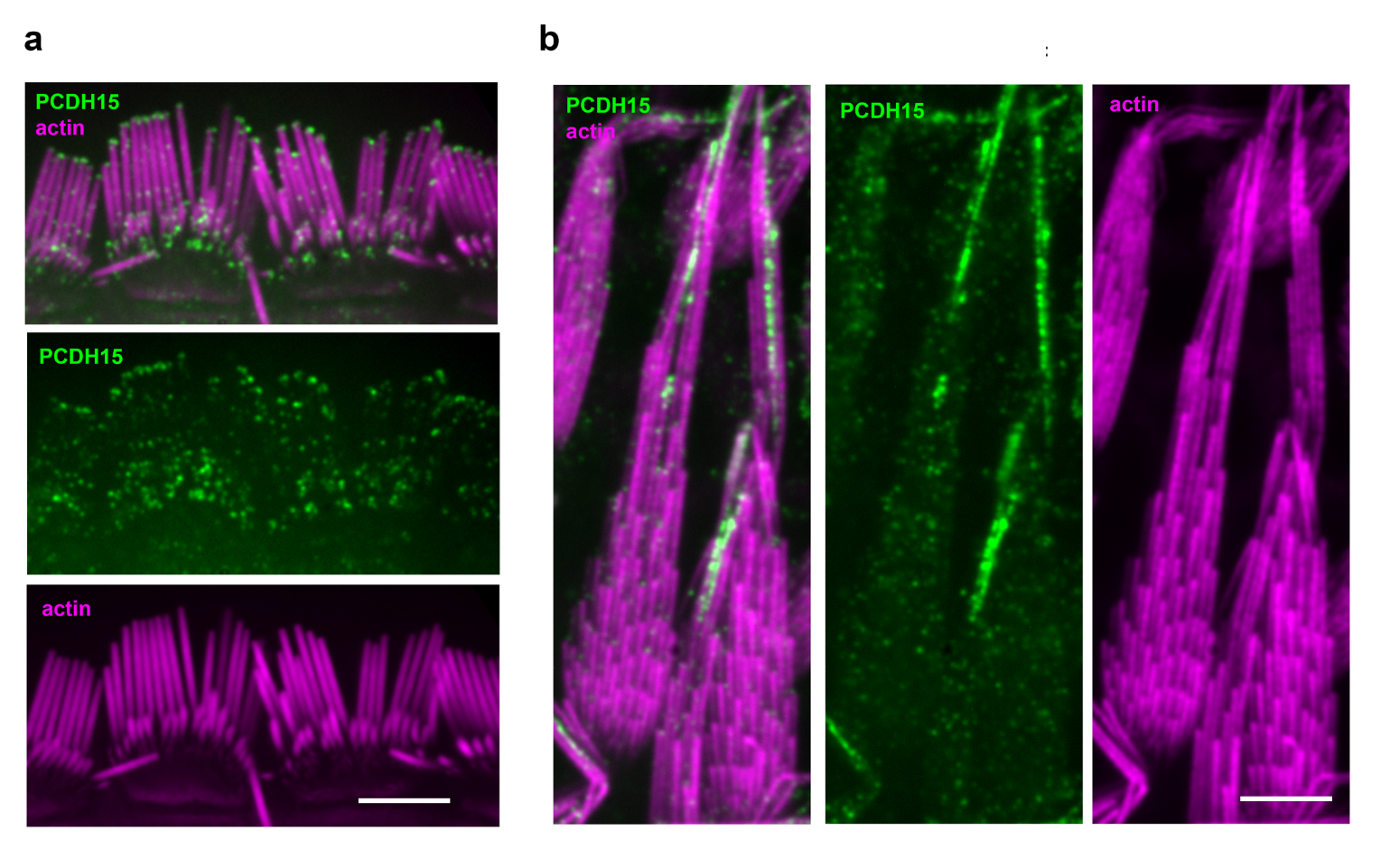


**Supplementary Figure 1: PDH15 localization in mammalian auditory and vestibular hair cells. a)** PCDH15 localizes to the tips of the stereocilia in 10-days old murine inner hair cells (IHC) as showed by immunohistochemistry with a pan PCDH5 antibody PB811 (green). **b**) PCDH15 (green) is accumulated at the kinociliary links in vestibular hair cells from adult guinea pig as showed by immunohistochemistry with a pan PCDH5 antibody. Tissue explants were counterstained with phalloidin to label F-actin and visualize the hair cell stereocilia (magenta). Scale bar = 5 μm.


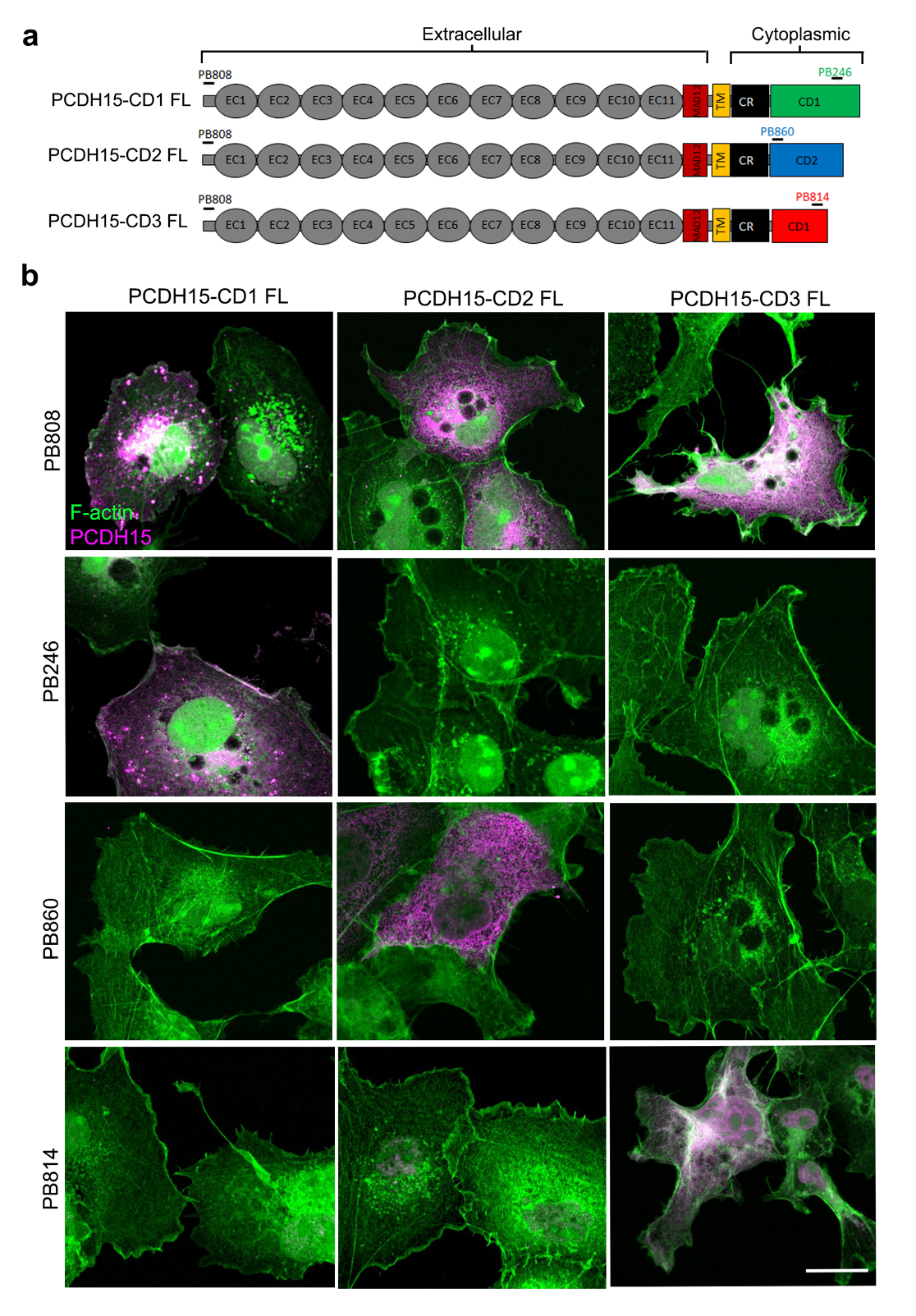


**Supplementary Figure 2: Validation of the anti PCDH15 antibodies and the Full-length PCDH15 constructs. a)** Diagram of the PCDH15-CD1, -CD2 and -CD3 constructs used in this study and localization of the peptides used to generate the PCDH15 antibodies PB808, PB246, PB860 and PB814. **b**) COS7 cells expressing any of the three PCDH15 isoforms (PCDH15-CD1, -CD2 or -CD3) were labeled with the pan-PDH15 antibody PB808, while only cells expressing the specific PCDH15 isoform were labeled with the isoform-specific anti PCDH15 antibody (magenta). Cells were counterstained with phalloidin to label F-actin (green). Scale bar = 15 μm.


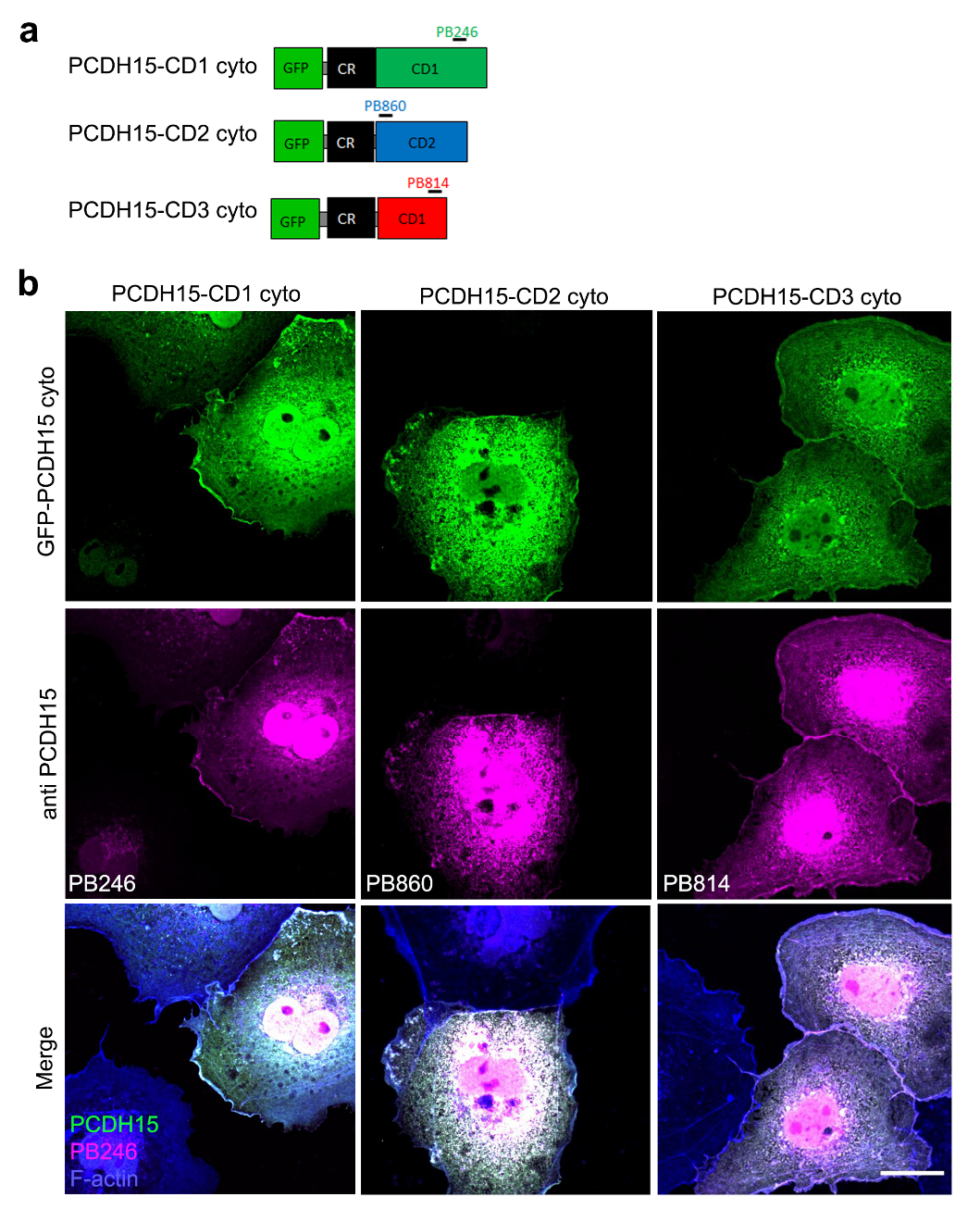


**Supplementary Figure 3: Validation of the anti PCDH15 antibodies and the cyto PCDH15 constructs. a)** Diagram of the cyto GFP-PCDH15-CD1, -CD2 and -CD3 constructs used in this study and localization of the peptides used to generate the isoform-specific PCDH15 antibodies PB246, PB860 and PB814. **b**) COS7 cells expressing the specific GFP tagged PCDH15 isoform (green) were also labeled with the corresponding isoform specific anti PCDH15 antibody (magenta). Cells were counterstained with phalloidin to label F-actin (blue). Scale bar = 15 μm.


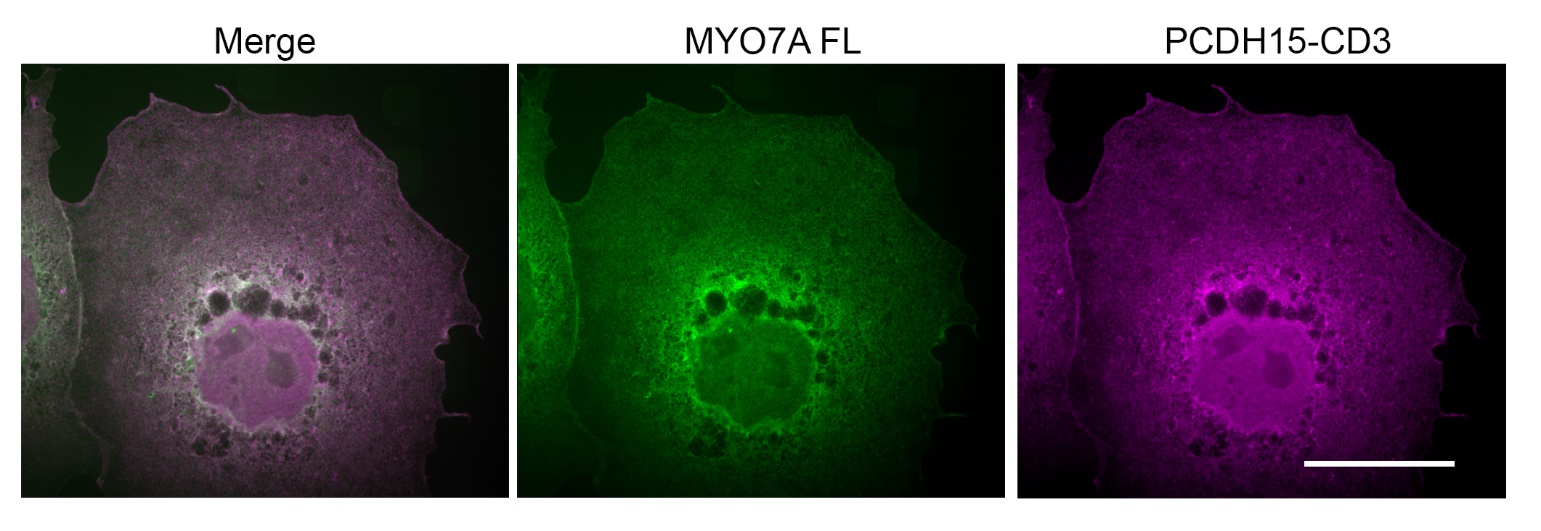


**Supplementary Figure 4: Myosin 7A does not form filopodia when expressed in COS7 cells.** COS7 cell expressing MYO7A full length (MYO7A FL) and PCDH15-CD3 shows that PCDH15-CD3 does not activate MYO7A. Scale bar = 15 µm.
